# The effects of race, gender, and alcohol cues on anger perception in crowds

**DOI:** 10.1080/00049530.2024.2426661

**Published:** 2025-01-19

**Authors:** Elizabeth Summerell, Liberty Shuttleworth, Carmen Lin, Thomas F. Denson

**Affiliations:** a School of Psychology, University of New South Wales, Sydney, Australia; b School of Psychology, University of Adelaide, Adelaide, Australia

**Keywords:** Anger, emotion recognition, crowds, race, alcohol

## Abstract

**Objective:**

Anger in crowds can be dangerous and lead to violence. Accurately assessing anger in crowds can be difficult, and people tend to overestimate the average intensity of a crowd’s anger relative to an individual’s anger (i.e. the crowd emotion amplification effect).

**Method:**

Across three experiments, we investigated the emotion amplification effect in crowds (versus individuals) displaying angry facial expressions. We also investigated the influence of gender, race, and alcohol cues as influences on this effect.

**Results:**

In two of the three experiments, we replicated the emotion amplification effect and found an interaction with race. Participants overestimated anger in White crowds more so than anger in Black crowds, but overestimated anger to a greater extent for Black individuals more than White individuals. There was also a main effect such that participants overestimated anger for men relative to women in both individuals and crowds and in both races.

**Conclusions:**

These findings highlight the bias to overestimate anger in White crowds, men, and Black individuals. These findings may affect policies around policing and crowd control.

## KEY POINTS

**What is already known about this topic:**

1. Anger in crowds can turn into violence.
2. People tend to overestimate the intensity of anger in crowds relative to individuals.
3. To date, only White faces have been used as stimuli to examine this overestimation effect.

**What this topic adds:**

1. White crowds elicited a greater overestimation effect than Black crowds.
2. Conversely, Black individuals elicited a greater overestimation effect than White individuals.
3. These findings may inform crowd control and policing strategies.

Humans have evolved the ability to quickly and accurately detect anger and other basic emotions in faces (Batty & Taylor, 2003; Hess & Thibault, 2009). Although other perspectives of emotion perception exist (e.g., Aviezer et al., 2017), emotional facial expressions can provide relatively accurate and clear information about people’s current emotions, thoughts, and intentions (Wieser & Brosch, 2012). For instance, facial anger displays elicit perceptions of strength, which can increase fear and bargaining power (Sell et al., 2014; Van Kleef et al., 2004).

When viewing laboratory analogues of crowds, it is thought that observers extract summary information about collective attributes in a process known as *ensemble coding* (Alvarez, 2011; Whitney & Yamanashi Leib, 2018; Whitney et al., 2014). It is generally accepted that observers are fairly accurate at extracting information about a crowd’s average emotion (Haberman & Whitney, 2007). However, a growing body of research finds that observers are particularly prone to display an anger bias when evaluating crowds (Goldenberg et al., 2021; Mihalache et al., 2021).

Crowds can be emotional. In certain crowd situations, such as protests, strikes, and political rallies, anger may be the dominant emotion in the crowd. Angry crowds are particularly worrisome because of the risk of erratic and destructive anger-fuelled behaviour. One paradigm that examines anger perception in crowds is the *crowd emotion amplification effect* (Goldenberg et al., 2021). This effect occurs when people estimate a crowd’s average emotion as more intense than it actually is. That is, overestimate the average intensity of a crowd’s emotion relative to individuals’ emotions.

To date, only a handful of experiments have directly investigated the crowd emotion amplification effect (e.g., Goldenberg et al., 2021). In this paradigm, participants are asked to judge the average emotion (e.g., anger or happiness) of a “crowd”. The crowd is operationalized as an array of 12 faces expressing mixed intensities of anger. A single face condition is used as a control. Results revealed that participants overestimated the crowd’s mean anger intensity to a greater extent than the individual faces. The crowd emotion amplification effect remained robust for long or short exposure times. Furthermore, varying the arrays from 1 to 12 faces produced a linear effect on amplification with smaller arrays showing less overestimation than larger arrays.

Researchers suggest that such systematic biases can be adaptive and may be employed to protect against greater anticipated threat, consistent with error management theory (D. D. Johnson et al., 2013; Mihalache et al., 2021). Error management theory suggests that humans have evolved a “a *bias* toward making the least costly error over time” (Johnson et al., 2013, p. 475). Error management theory has been applied to a broad range of decision-making phenomena. Such a bias is observed for crowds when people overestimate anger relative to individuals. Crowds are not inherently more dangerous than individuals, yet crowds do hold the potential to overpower perceivers via greater physical presence. Thus, when faced with crowds displaying some degree of anger, it may be adaptive to overestimate the amount of anger in the crowd. Doing so may initiate self-protective behaviour in observers. In the present research, we manipulated three social factors that could exacerbate the perceived dangerousness of a crowd: gender, race, and the presence of alcohol.

Understanding the influence of features of crowd settings on anger judgements in crowds has important implications for public safety, intergroup conflict, and applied settings. However, little is known about factors that influence the crowd emotion amplification effect. The present research aimed to address this gap by investigating three novel, theoretically relevant factors: race, gender, and alcohol cues. One strength of the present research is the ability to distinguish which of these factors occurs at the crowd level, individual level, neither level, or both levels. To this end, we investigated main effects and interactions among the manipulations on the overestimation effect.

## Race

The extant emotion amplification research has almost exclusively used arrays of White men as stimuli. However, there is good reason to suggest that race may impact the crowd emotion amplification effect. In Western, Educated, Industrialized, Rich, and Democratic contexts (WEIRD, Henrich et al., 2010), Black people are frequently the targets of harmful stereotypes portraying them as threatening (Becker et al., 2010, Chiricos et al., 2004; Cottrell & Neuberg, 2005; Devine & Elliot, 1995; Hugenberg, 2005; Najdowski et al., 2015). Anger is more readily identified in Black faces than in White faces (Hugenberg, 2005), and Black individuals are more likely to be misperceived as angry (Halberstadt et al., 2018; Hutchings & Haddock, 2008). In a test of the crowd emotion amplification effect, participants viewed crowd arrays with varying proportion of Black and White faces (Goldenberg et al., 2022). Participants rated the arrays with relatively more Black faces as more emotional in terms of happiness and anger.

Together, consistent with error management theory, this research suggests the anger amplification effect may be enhanced for crowds of all Black faces relative to crowds of all White faces. Thus, we should observe a spreading interaction such that the amplification effect is much larger for groups of Black people than White people or Black individuals. We acknowledge that racial and ethnic stereotypes likely vary as a function of culture and environment (see Fiske, 2017; D. J. Johnson & Chopik, 2019; Somo et al., 2021). Consequently, we restrict our conclusions to other WEIRD countries.

## Gender

In addition to race, we investigated whether crowds of men would produce a larger overestimation of anger than arrays of women. From an early age, people learn to associate anger with “maleness” (Birnbaum, 1983; Birnbaum et al., 1980), including at the implicit level (Becker et al., 2007). When individual faces are clearly presented as angry, people are quicker and more accurate at identifying anger in men than women (Becker et al., 2007). Neutral male faces are judged as more angry than neutral female faces (Adams et al., 2012) and angry faces are more likely to be categorized as male (Bayet et al., 2015). Male faces are also more likely than female faces to be misperceived as angry when flanked by angry faces (Neel et al., 2012). Anger displays are judged as more appropriate for males, a finding that is partially explained by perceived dominance (Hess et al., 2005). Groups of men commit much of the world’s extreme acts of violence such as violent raids, riots, and warfare and hold more power over groups of women and society (Van Vugt, 2009). Thus, we predicted that arrays of male faces would produce a larger amplification effect than arrays of female faces. As with race, this effect would be observable in a spreading interaction, with groups of men eliciting a greater overestimation effect than groups of women and individuals.

## The presence of alcohol

Alcohol is present in many crowd settings (e.g., concerts, sporting events, riots, nightclubs; Moore et al., 2008; Ostrowsky, 2018). Intoxicated people can be threatening. For instance, in one year, 13% of Australians reported feeling afraid of intoxicated people (Morgan & McAtamney, 2009). However, research has not yet examined the impact of alcohol-related cues on emotion amplification in individuals or crowds. Alcohol priming increases activation of alcohol-related concepts, which increased aggression-related cognition (Bartholow & Heinz, 2006) and aggressive behaviour (Pedersen et al., 2014; Subra et al., 2010). In light of these alcohol priming effects, it is likely that a perceiver’s ability to “read” a crowd may be further affected by exposure to alcohol-related cues. Exposure to alcohol-related images can also increase social disinhibition (Freeman et al., 2010) and race bias (Stepanova et al., 2012). Indeed, alcohol cues and alcohol intoxication can increase hostility towards Black and Middle Eastern people (Schofield et al., 2017; Stepanova et al., 2018).

## The present research

As the crowd emotion amplification effect is new, replication studies are needed to confirm the effect and investigate additional possible influences. It is currently unclear if emotion amplification is influenced by the race or gender of the crowd, or the presence of alcohol-related cues. Here we test whether individuals overestimate the average intensity of a crowd’s emotion (i.e., the crowd emotion amplification effect) to a greater extent relative to individuals’ emotions. That is, Experiment 1 aimed to determine whether estimates of emotion intensity would be significantly higher for facial arrays containing 12 faces, compared to facial arrays containing a single face and identify alcohol cues and gender as potential moderators of this effect.

We preregistered the following hypotheses (https://osf.io/2yr68/). First, we expected estimates of emotion intensity to be significantly higher for facial arrays containing 12 faces, compared to facial arrays containing a single face. Second, we predicted interactions of facial array size (i.e., 12 faces versus one face) with gender and alcohol cues such that male gender and exposure to alcohol cues would increase the degree of crowd emotion amplification. That is, crowds of men, and cues to intoxicated crowds would elicit greater anger perception their individual and group counterparts. Such groups may be perceived as more threatening and hence, angrier than individuals.

In Experiments 2 and 3, we also predicted an interaction of facial array size (i.e., 12 faces versus one face) with race, such that Black race would increase the degree of crowd emotion amplification. That is, crowds of Black people would elicit greater anger perception than their individual and group counterparts. We also predicted an alcohol cue × race interaction, such that alcohol cues would have the largest effect on amplification for the Black faces relative to the White faces.

Participants saw each face an average of 14 times over 168 trials. Although the exact number of trials within each type was randomly determined, approximately half of the trials were individuals and half were groups; half were women and half were men; in Experiments 2 and 3, half had Black faces and half had White faces.

## Ethics statement, power analysis

All studies were approved by the Human Research Ethics Advisory Panel C (#3373). Given the initial sample of 50 (Goldenberg et al., 2020), and the need for larger sample sizes in replication studies, we aimed to recruit 150 participants per experiment. Although we initially used GPower in the preregistration, we subsequently realized that the *simr* package (Green et al., 2016) in R is more appropriate for calculating power for linear mixed effects models. We calculated power based on our preregistered sample size of 150 participants and the fixed and random effects parameters from Experiment 1. Using 1000 simulations, we subsequently tested for power of all fixed main effects and interactions at three different unstandardized slopes, *b*s = 0.50, 0.75, and 1.00. These putative effect sizes could arguably be considered quite small as even a main effect of *b* = 1.00 would translate into a 1-point difference in anger ratings between conditions on a 50-point scale. These simulations showed that power ranged between 65% and 100% for main effects, 42% to 93% for two-way interactions, and 23% to 65% for three-way interactions (see Table 1). While these values indicate that main effects and two-way interactions are sufficiently powered within the current experiments, we acknowledge that these experiments are likely underpowered for higher-order interaction effects.

**Table 1.** Power analysis for each regression estimate across three different effect sizes.

Regression Slopes	Effect sizes		
	0.50	0.75	1.00
Size	66%	94%	100%
Gender	66%	95%	100%
Alcohol	66%	94%	100%
Size × Gender	42%	73%	93%
Size × Alcohol	44%	76%	93%
Alcohol × Gender	45%	76%	93%
Size × Alcohol × Gender	23%	42%	65%

Analyses were conducted in the *simr* package with 1000 simulations for each estimate.

**Figure 1.**
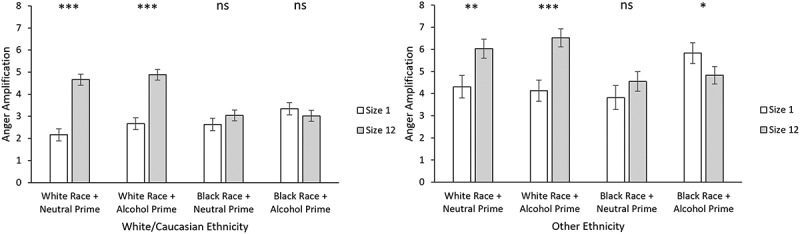
Anger amplification as a function of size, drink, crowd race, and ethnicity. Participants in the left panel were of White/Caucasian ethnicity. Participants in the right panel identified as another ethnicity.

We note here a deviation from our preregistration. In addition to our planned analyses, we ran a supplementary exploratory analysis to determine whether the ethnicity of participants would influence the amplification effect. For simplicity, we examined participant ethnicity by comparing White participants compared to all other ethnicity categories combined (i.e., Other). Ethnicity was added as an interaction term (dummy coded with White as the reference group). No significant relationships between participant ethnicity and the crowd emotion amplification effect were observed in Experiments 1 and 3. However, in Experiment 2, we observed a size × drink × crowd race × ethnicity interaction (see Figure 1). To investigate this, data were grouped according to participant ethnicity (White v Other), and linear mixed effects models were run on each group. Size, drink, and crowd race were specified as fixed factors, and participant was specified as a random factor. For White participants, we observed a significant size × crowd race interaction, *b* = 2.32, *SE* = 0.46, *t*(12248.98) = 5.03, *p* < .001. Contrast analysis revealed that amplification was greater for White crowd trials compared to White single trials (contrast estimate = −2.37, SE = 0.23, *z* = −10.24, *p* < .001). Amplification did not differ between Black crowd trials compared to Black single trials (contrast estimate = −0.06, *SE* = 0.23, z = −0.27, *p* = .791). For participants who identified as other than White, we observed a significant size × drink × crowd race interaction, *b* = −2.67, *SE* = 1.09, *t*(4730.35) = −2.46, *p* = .014. Contrast analysis revealed amplification was greater for Black individuals compared to Black crowds, when preceded by alcohol primes (contrast estimate = 1.35, *SE* = 0.54, *z* = 2.50, *p* = .012). Amplification did not differ between Black individuals compared to Black crowds when preceded by neutral primes (contrast estimate = −0.71, *SE* = 0.55, *z* = −1.29, *p* = .197). Amplification was greater for White individuals compared to White crowds when preceded by both alcohol primes (contrast estimate = −2.24, *SE* = 0.53, *z* = −4.21, *p* = < .001) and neutral primes (contrast estimate = −1.63, *SE* = 0.55, *z* = −2.98, *p* = .003).

## Experiment 1

### Method

The aim of Experiment 1 was to replicate the crowd emotion amplification effect and test the roles of two moderators on this effect (i.e., gender and alcohol cues).

#### Participants

A total of 159 American Mechanical Turk workers took part in the experiment in exchange for USD$2.45. Participants were also required to have a worker approval rating of at least 95% based on at least 100 previous HITs. Data from 14 participants were excluded for incomplete responses and/or failing both attention checks.^1^ The final sample comprised 145 participants (42 women; *M*_*age*_ = 37.46, *SD* = 11.11). Their reported ethnicities were: White (68.97%), Asian (14.48%), Black (8.27%), Hispanic/Latino/Latina (4.83%), Indian (1.38%), American Indian/Alaskan Native (0.69%), and multiple ethnicities (1.38%). The highest level of education was: Year 11 or 12 (17.93%), Bachelor’s degree (53.10%), Associate degree (13.10%), Postgraduate degree (15.17%), prefer not to answer (0.69%).

#### Design and procedure

**Figure 2.**
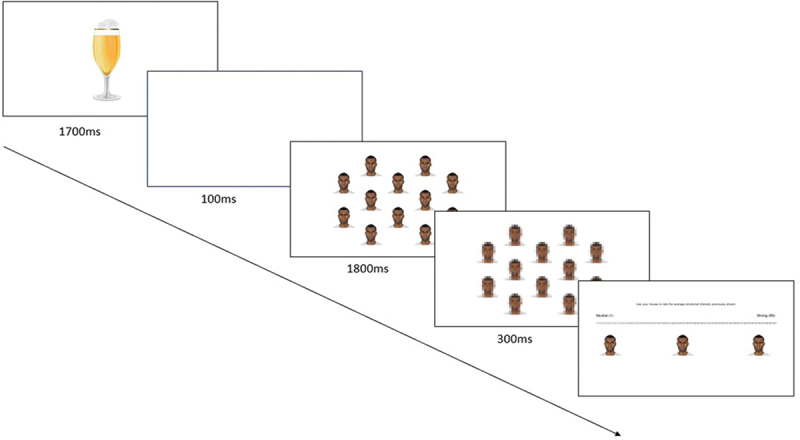
An example trial containing an alcoholic image prime, and an array of 12 African American male faces.

The design closely resembled Goldenberg et al. (2020) and was administered using Inquisit (www.millisecond.com). To access and complete the study, participants were required to be on a standard desktop computer or laptop and install the Inquisit 5 Player web application. Participants provided informed consent and demographic information before completing two practice trials and 168 test trials. For all trials, participants viewed an image of an alcoholic or a non-alcoholic beverage for 1700 ms, followed by a blank screen for 100 ms. Participants then viewed an array of one or twelve faces expressing different intensities of anger for 1800 ms (see Figure 2 for a breakdown of trial components). As in Goldenberg et al. (2020), the same face was presented for each trial, but the anger intensity of the face(s) varied. For each trial, the mean anger intensity, set size (1 or 12 faces), gender (men or women), and alcohol cues (alcoholic or non-alcoholic drink) were randomized. Participants were then presented with masks in the same location as each face array for 300 ms.^2^ Next, for the dependent measure, participants indicated the perceived anger level expressed in the group by moving their mouse along a scale from 1 (*neutral*) to 50 (*extremely angry*). Three images were anchored below the appropriate location along the scale at 1, 25, and 50. To calculate the emotion amplification effect, we computed the difference between estimates of the crowd mean emotional intensity and the actual crowd mean. Following the task, participants completed measures of trait aggression and alcohol use.

#### Materials

##### Face images

**Figure 3.**
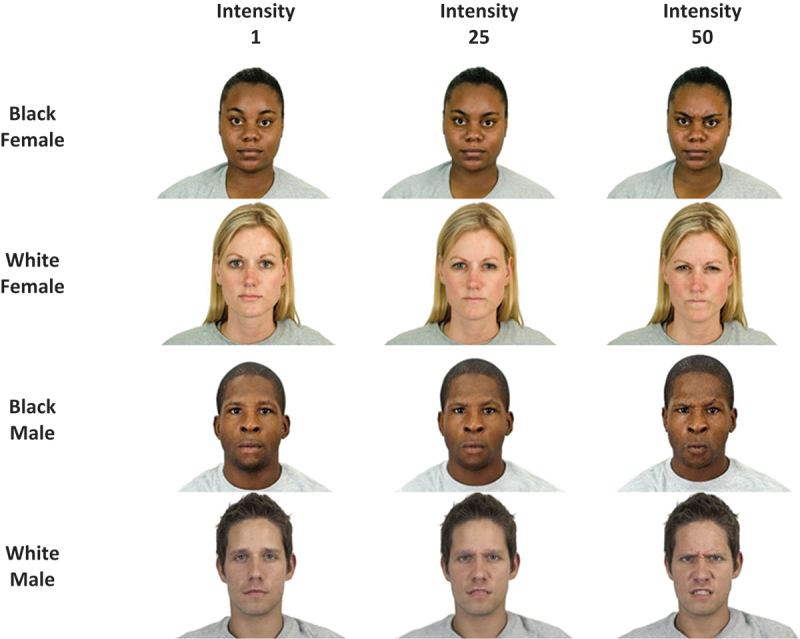
Examples of all four identity categories (gender × race), at intensities 1, 25, and 50. Experiment 1 used only images of white males and white females. Experiments 2 and 3 used all image categories.

Angry and neutral faces were sourced from the Chicago Faces Database (Ma et al., 2015). All faces were forward-looking and appeared to be making eye-contact with the observer. A total of 14 different faces were used: seven White male faces, and seven White female faces. Images were morphed from neutral to angry on a 50-point scale from 1 (*neutral*) to 50 (*extremely angry*), using FantaMorph (Abrosoft FantaMorph Version 5.4.2). For examples of stimulus categories across anger intensities, see Figure 3 For each trial, the mean anger intensity was randomly assigned between 10 and 40 from a uniform distribution (1 = neutral, 50 = extremely angry). The “true” anger value was based on the numeric value underlying the morphed face. So, for instance, for a median level of anger on an individual trial, the true value for that trial was 25, which would be a halfway morph from no anger to full anger. For the crowd trials, the true values were averaged for all 12 faces. Emotion amplification was calculated by subtracting this “actual mean intensity” from participants’ ratings of anger intensity.

##### Alcohol images

Standardized images of alcoholic and non-alcoholic beverages were sourced from the American Alcohol Photo Stimuli database (Stauffer et al., 2016). Images were matched for complexity, form, and colour to the best extent possible.

##### Trait aggression

The Brief Aggression Questionnaire (BAQ: Webster et al., 2014) is a 12-item self-report that assesses four dimensions of trait aggression: physical aggression, verbal aggression, anger, and hostility. As noted in the preregistration, we analysed the total score rather than individual subscales. The BAQ has good convergent and discriminant validity, and internal and test-retest reliability (Webster et al., 2015).

##### Alcohol use

The Alcohol Use Disorders Identification Test (AUDIT: Babor et al., 2001) is a 10-item self-report questionnaire developed by the World Health Organization to assess alcohol consumption and dependence. As noted in the preregistration, we used the total score in our analysis. The AUDIT has good convergent and discriminant validity, and internal and test-retest reliability (de Meneses-Gaya et al., 2009; Reinert & Allen, 2007).

### Statistical analyses

All analyses were conducted in R (R Core Team, 2021). Linear mixed effects models were analysed with the *lme4* package (Douglas et al., 2015); 95% confidence intervals (CIs) were calculated with the *lmerTest* package (Kuznetsova et al., 2017); *R*^*2*^ was calculated with Nakagawa et al. (2013) method from the *performance* package (Lüdecke et al., 2021). We specified participant as a random effect and the crowd size, crowd gender, the presence of alcoholic cues, and their interactions as fixed effects. A secondary analysis included trait aggression and alcohol use as fixed effects. As indicated in our preregistration, outliers defined as having residuals > |3 *SD*s| were omitted from analyses, which resulted in removal of 0.60% (136 trials) of the data.

## Results and discussion

### Primary analyses

**Figure 4.**
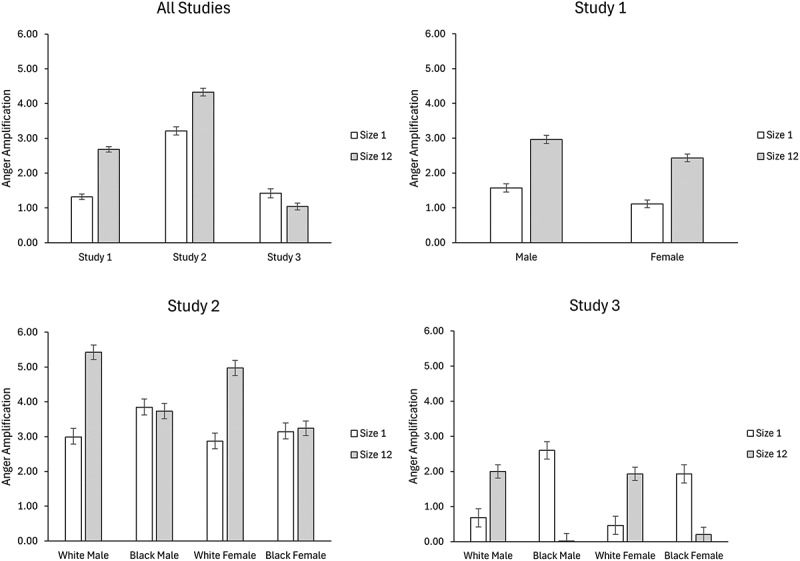
Mean anger amplification scores as a function of study (panel 1), gender (panel 2; study 1), and race × gender (panels 3 and 4; studies 2 and 3).

Main effects and interactions were sequentially entered from Steps 1–3. Replicating the crowd emotion amplification hypothesis, perceived anger was overestimated by 1.36 points (scale from 1 to 50) for trials containing 12 faces compared to trials with one face, *b* = 0.12, *SE* = 0.01, *CI*_95_ [0.11, 0.14], *t*(22349.32) = 13.18, *p* < .0001 (Table 2; Figure 4). Participants also overestimated the anger intensity of trials with men’s by 0.50 points compared to women’s faces, *b* = 0.50, *SE* = 0.10, *CI*_95_ [0.30, 0.71], *t*(22349.15) = 4.88, *p* < .0001. Contrary to our hypotheses, alcohol-related cues did not affect emotion amplification, nor were there any further significant predictors. The model explained 18.5% of the variance in crowd emotion amplification. No significant two- or three-way interactions were observed.

**Table 2.** Impact of crowd size, crowd gender, presence of alcoholic cues, crowd race, and their interactions on anger amplification.

	*Experiment 1* (22,497 trials, *N* = 145)				*Experiment 2* (17,102 trials, *N* = 110)				*Experiment 3* (21,740 trials, *N* = 140)			
	b	SE	95% CI	t-value	b	SE	95% CI	t-value	b	SE	95% CI	t-value
Step 1												
Size	0.12	0.01	0.11, 0.14	13.18***	1.10	0.14	0.82, 1.37	7.84***	−0.40	0.16	−0.71, −0.09	−2.50*
Drink	0.02	0.10	−0.18, 0.23	0.24	−0.38	0.14	−0.66, −0.11	−2.74**	0.09	0.16	−0.22, 0.40	0.57
Crowd gender	0.50	0.10	0.30, 0.71	4.88***	−0.37	0.14	−0.65, −0.10	−2.65**	0.18	0.16	−0.13, 0.49	1.12
Crowd race	–	–	–	–	0.50	0.14	0.23, 0.78	3.59***	0.09	0.16	0.22, 0.40	0.57
Step 2												
Size × drink	0.01	0.02	−0.03, 0.04	0.43	0.56	0.28	0.02, 1.11	2.02*	0.46	0.32	0.16, 1.08	1.46
Size × crowd gender	0.01	0.02	−0.03, 0.04	0.41	−0.13	0.28	−0.68, 0.42	−0.46	0.48	0.32	−0.14, 1.10	−1.50
Drink × crowd gender	−0.03	0.21	−0.44, 0.38	−0.15	−0.003	0.28	−0.55, 0.54	−0.01	−0.22	0.32	−0.84, 0.40	−0.70
Size × crowd race	–	–	–	–	2.29	0.28	1.75, 2.84	8.22***	3.46	0.32	2.84, 4.08	10.93***
Drink × crowd race	–	–	–	–	0.23	0.28	−0.32, 0.77	0.82	0.27	0.32	−0.35, 0.89	0.85
Crowd gender × crowd race	–	–	–	–	0.35	0.28	−0.19, 0.90	1.27	0.08	0.32	−0.54, 0.70	0.24
Step 3												
Size × drink × crowd gender	0.06	0.04	−0.01, 0.13	1.61	−0.15	0.56	−1.25, 0.94	0.78	0.06	0.63	−1.18, 1.30	0.10
Size × drink × crowd race	–	–	–	–	−0.76	0.56	−1.86, 0.33	−0.17	−0.30	0.63	−1.54, 0.94	−0.48
Size × crowd gender × crowd race	–	–	–	–	−0.78	0.56	−1.87, 0.31	−1.40	−0.53	0.63	−1.77, 0.71	−0.83
Drink × crowd gender × crowd race	–	–	–	–	−0.44	0.56	−1.54, 0.65	−0.79	0.39	0.63	0.85, −1.63	0.62
Step 4												
Size × drink × crowd gender × crowd race	–	–	–	–	−0.27	1.12	−2.46, 1.92	−0.24	0.92	1.27	−1.56, 3.40	0.73
	*R*^*2*^_*participants*_ = .178, *R*^*2*^_*fixed effects*_ = .007				*R*^*2*^_*participants*_ = .260, *R*^*2*^_*fixed effects*_ = .007				*R*^*2*^_*participants*_ = .052, *R*^*2*^_*fixed effects*_ = .006			

* = *p* < .05; ** = *p* < .01; *** = *p* < .001.

### Exploratory analyses

The model adding trait aggression and alcohol use as fixed effects showed a significant relationship between alcohol use and emotion amplification, *b* = 0.21, *SE* = 0.04, 95% CI95 [0.13, 0.29], *t*(141.95) = 5.10, *p* < .0001; but this relationship did not differ for crowds or individuals. This finding suggests that heavier alcohol users showed a tendency to overperceive anger more than lighter drinkers in both crowds and single faces.^3^ Trait aggression was not related to emotion amplification, *b* = .04, *SE* = .03, 95% CI [−0.02, 0.10], *t*(141.86) = 1.19, *p* = .236.^4^ Participant ethnicity was not related to emotion amplification.

## Experiment 2

Experiment 1 replicated the crowd emotion amplification effect such that an array of twelve faces elicited a greater overestimation of perceived anger than individual faces. We also identified two novel factors influencing perceived anger: gender and prior risky drinking behaviour. Specifically, participants overestimated anger in men’s faces to a greater extent than women’s faces, regardless of whether they viewed one or twelve faces. We also found that heavy drinkers showed a heightened amplification effect to both individuals and crowds relative to lighter drinkers. However, the alcohol cues presented prior to viewing the faces did not influence the amplification effect. Experiment 2 aimed to replicate and extend findings from Experiment 1. In addition, Experiment 2 also included a manipulation of race (i.e., Black American faces versus White American faces) with the expectation that Black faces would elicit a heightened anger crowd amplification than White faces.

## Method

### Participants

A total of 150 American participants from Mechanical Turk completed the experiment in exchange for USD$2.45. Data from 19 participants were lost due to technical issues, and data from participants were omitted for failing both attention checks (*n* = 3), or not completing the survey (*n* = 17), or insufficient data (*n* = 1). The final sample comprised 110 participants (43 women, 1 prefer not to disclose; *M*_age_ = 34.86, *SD* = 9.38). Their reported ethnicities were: White (71.82%), Black (20.91%), Asian (4.54%), Hispanic/Latino/Latina (0.91%), Multiple ethnicities (0.91%), and Prefer not to disclose (0.91%). The highest level of education was: Year 11 or 12 (9.01%), Bachelor’s degree (52.73%), Associate degree (9.01%), or Postgraduate degree (29.10%).t(8469.86) = 0.52, *p* = .60. We removed 0.72% (124 trials) of the data based on our outlier criterion.

### Design, materials, and procedure

The experiment design is identical to Experiment 2 with the addition of stimuli varying in race (i.e., White or African American) and was administered online through Inquisit. Participants viewed twelve individual identities, three from each gender × race category (i.e., three Black female faces, three White female faces, three Black male faces, three White male faces; see Figure 3 for examples). Participants viewed only a single identity on any given trial.

## Results and discussion

### Primary analyses

Replicating the crowd emotion amplification hypothesis, perceived anger was overestimated by 1.13 points (scale 1 to 50) for trials containing 12 faces than trials with one face, *b* = 1.10, *SE* = 0.14, *CI*_95_ [0.82, 1.37], *t*(16989.50) = 7.84, *p* < .001 (Table 2). Perceived anger was overestimated by 1.19 points for trials with men’s faces compared to trials with women’s faces, *b* = −0.37, *SE* = 0.14, *CI*_95_ [−0.65, −0.10], *t*(16989.99) = −2.65, *p* = .008. Perceived anger was overestimated by 0.43 points for trials preceded by alcohol primes compared to trials preceded by neutral primes, *b* = −0.38, *SE* = 0.14, *CI*_95_ [−0.66, −0.11], *t*(16990.20) = −2.74, *p* = .006. Contrary to our hypothesis, perceived anger was overestimated by 0.56 points for trials with White faces compared to trials with Black faces, *b* = 0.50, *SE* = 0.14, *CI*_95_ [0.23, 0.78], *t*(16989.59) = 3.59, *p* = < .001.

We observed a size × drink interaction. Follow-up analysis showed that when faces were presented individually, perceived anger was overestimated by 0.72 points for trials preceded by alcohol primes than trials preceded by neutral primes, *b* = −0.67, *SE* = 0.21, *CI*_95_ [−1.09, −0.26], *t*(8413.51) = −3.18, *p* = .002. However, in trials with 12 faces perceived anger did not differ significantly as a function of prime, *b* = −0.09, *SE* = 0.18, CI95 [−0.45, 0.26], *t*(8469.86) = −0.53, *p* = .600.

We also observed a size × crowd race interaction. Follow-up analysis showed that when faces were presented individually, participants overestimated anger by 0.57 points in Black individuals compared to White individuals, *b* = 0.66, *SE* = 0.21, CI_*95*_ [0.24, 1.07], *t*(8411.51) = 3.11, *p* = .002; however, this effect was reversed in the trials with 12 faces (Table 2; Figure 4). Specifically, participants overestimated anger by 1.71 points in White crowds compared to Black crowds, *b* = −1.65, *SE* = 0.18, CI_*95*_ [−2.00, −1.29], *t*(8468.92) = −9.14, *p* < .0001. The three-way interactions and four-way interaction were not significant. The model explained 26.7% of the variance in crowd emotion amplification.

In sum, Experiment 2 replicated two main effects from Experiment 1: the crowd amplification effect and the finding that men were perceived as showing greater anger than women. We introduced race as a new manipulation and found that Black faces were perceived as angrier than White faces but only when presented individually. White people were perceived as angrier when presented as a crowd. We also found that priming with alcohol-related cues increased anger overestimation, but this effect was confined to trials with individual faces.

### Exploratory analyses

As in Experiment 1, we examined whether trait aggression and alcohol use would be associated with the emotion amplification effect. Neither trait aggression, *b* = 0.12, *SE* = 0.06, CI_*95*_ [−0.005, 0.25], *t*(106.94) = 1.88, *p* = .063, or alcohol use were significant predictors, *b* = 0.12, *SE* = 0.06, CI_*95*_ [−0.007, 0.25], *t*(106.94) = 1.85, *p* = .068.

## Experiment 3

Experiment 2 replicated and extended findings Experiment 1. The aim of Experiment 3 was to conduct a direct replication of Experiment 2 and conceptual replication of Experiment 1.

### Participants

Participants were 150 undergraduate Psychology students at the University of New South Wales who participated in exchange for partial course credit. Data from 7 participants were excluded for failing both attention checks or not completing the survey. The final sample comprised 143 participants (93 women, 1 preferred not to answer; *M*_age_ = 19.31 (SD = 2.07). Their reported ethnicities were: Asian (58.62%), White (21.38%), Indian subcontinent (6.21%), Middle Eastern (3.45%), Multiple ethnicities (7.59%), Prefer not to disclose (1.38%). We removed of 0.33% (72 trials) of the data based on our preregistered criterion for outliers.

### Design, materials, and procedure

The experiment design is identical to Experiment 2 and was administered online through Inquisit. All measures were identical to those used in Experiment 2.

## Results and discussion

### Primary analyses

Of the four manipulations, only the crowd manipulation significantly influenced emotion amplification, *b* = −0.40, *SE* = 0.16, *CI*_95_ [−0.71, −0.09], *t*(21609.54) = −2.50, *p* = .013 (Table 2; Figure 4). Unexpectedly, the reverse effect was observed. Participants overestimated anger by 0.38 points on trials containing one face relative to trials with 12 faces. Although contrary to our hypotheses, this finding is partially consistent with recent evidence. One experiment found that that anger amplification was greater for individuals than crowds, but only when anger intensity was high (Mihalache et al., 2021). Thus, emotional intensity may moderate the effect of size on the crowd emotion amplification effect.

The follow-up analysis for the size × crowd race interaction showed the same pattern of overestimation as that observed in Experiment 2. Specifically, when faces were presented individually, participants overestimated anger by 1.70 points in Black individuals compared to White individuals, *b* = 1.66, *SE* = 0.25, CI_95_ [1.17, 2.16], *t*(10619.24) = 6.59, *p* < .0001; however, this effect was reversed in the trials with 12 faces (Figure 4). Specifically, participants overestimated the anger by 1.86 points in White crowds compared to Black crowds, *b* = −1.79, *SE* = 0.19, CI_95_ [−2.17, −1.42], *t*(10884.66) = −9.31, *p* < .0001. None of the other interactions were significant. The primary model explained 5.2% of the variance in crowd emotion amplification.

The size × alcohol interaction that we observed in Experiment 2 was not observed here. One possibility for this occurrence is that the beverage images were American. Some of the brand names are not sold in Australia. Thus, Australians may not have had the same associations of anger and alcohol with these stimuli.

By contrast, it may be surprising that the American and Australian samples both showed the size × crowd race interaction. Indigenous (i.e., Black) Australians suffer many of the same stereotypes that African Americans do (e.g., aggressive, prone to criminality). Like African Americans, their welfare is far below that of White Australians in terms of life expectancy, poverty, alcoholism, physical health, and education. Australians show an implicit bias in favour of Caucasian faces compared to Indigenous faces that is comparable to US residents’ bias in favour of Caucasian faces compared to African American faces (Shirodkar, 2019). Further, a UN Human Rights Council investigation reported about Australia: “ … people of African descent face racial profiling, racial slurs, abuse of authority, over policing, under protection, targeting and violence” (UN OHCHR, 2022). Thus, the results of Experiment 3 May reflect a generalizing of this bias to Black faces regardless of ethnicity. Alternatively, Australians and Americans may simply hold similar negative stereotypes about African Americans, perhaps due to widespread exposure to media and social media. The significant race × size interactions from Experiments 2 and 3 (those that included race) suggest that either or both of these possibilities are plausible.

### Exploratory analyses

As in the previous experiments, we examined whether trait aggression and alcohol use would be associated with the emotion amplification effect. Neither trait aggression, *b* = 0.06 *SE* = 0.03, CI_*95*_ [−0.003, 0.12], *t*(135.42) = 1.80, *p* = .074, or alcohol use met conventional levels of significance, *b* = 0.08, *SE* = 0.04, CI_*95*_ [−0.08, 0.21], *t*(149.00) = 1.90, *p* = .06.

## General discussion

People can accurately recognize anger observed on individual faces (e.g., Ekman et al., 1987); yet perceivers tend to overestimate the extent of anger in groups (Goldenberg et al., 2021). Understanding how social factors may influence this finding, known as the crowd emotion amplification effect, was the impetus of the present research. We conducted three experiments involving over 60,000 trials and 395 participants to replicate the effect and manipulate potential moderators: alcohol cues, gender, and race. We observed inconsistent evidence of the crowd emotion amplification effect. Specifically, participants overestimated anger in crowds of 12 faces to a greater extent than individual faces in Experiments 1 and 2, but not Experiment 3.

Although we have couched our experiments in evolutionary theory of emotion perception, it is equally plausible that contextual models can explain our data (e.g., Aviezer et al., 2017). These models show that context can influence emotion perception and can even override the effects of basic facial emotion displays. Thus, participants may have responded with greater anger perception to the crowds because the context is different than lone individuals. That is, if many people are relatively angry, the context suggests that there is something to be truly angry about, and hence may have elicited an overestimation effect. This explanation is consistent with the findings of Experiments 1 and 2 but does not explain the reversed effect of size in Experiment 3. In fact, the reversed effect in Experiment 3 questions the universality of the crowd emotion amplification effect. The sample in Experiment 3 was a group of primarily Asian Australian undergraduates; whereas, MTurk participants comprised the samples in Experiments 1 and 2. The levels of amplification were also lower in Experiment 3 than in the previous two experiments, suggestion that the manipulation may have had less impact on anger perception in the Australian student sample. Future research could examine the universality of the effect.

### Potential moderators

#### Race

The search for moderators of the crowd emotion amplification effect was partially successful. Of the three potential moderators (i.e., race, gender, and alcohol cues) the only robust moderator of the crowd emotion amplification effect was race, although the nature of this interaction differed from our pre-registered hypothesis. We had expected participants to overestimate anger for Black crowds to a greater extent than White crowds due to associations of Black people with threat (Becker et al., 2010; Cottrell & Neuberg, 2005; Devine & Elliot, 1995; Hugenberg, 2005). However, individual Black faces were rated as angrier than a “crowd” of 12 Black people. By contrast, White faces were rated as angrier in crowds than as individuals. Thus, at the individual level, we replicated the common finding that Black people are stereotyped as more aggressive than White people (D. J. Johnson & Chopik, 2019; Motro et al., 2022). The unusual effect was at the crowd level, in which White people were rated as overly angry compared to crowds of Black people in Experiments 2 and 3.

In accounting for the observed perceptions of Black crowds, one possibility is that observers in our study were overcorrecting for prejudice in their ratings of Black faces in crowds (e.g., Lloyd et al., 2017; Lloyd & Hugenberg, 2021; Plant & Devine, 1998). For example, past research finds that observers divert their gaze away from Black faces to avoid appearing prejudiced (Richeson & Trawalter, 2008). Future research employing study designs that allow for tests of potential mechanisms, such as eye tracking, may be beneficial in explaining this pattern of results.

In accounting for the observed perceptions of White crowds, one possibility may be a change in the stereotypes and prejudices of Americans due to the political climate in the United States at the time of data collection (2020–2021). That is, a decline in pro-White sentiment following the political emergence of Donald Trump and the rise of the Black Lives Matter movement. Research suggests that during Black Lives Matter protests, overall implicit pro-white attitudes became less positive (Sawyer & Gampa, 2018). Similarly, following Trump’s emergence as a political candidate, White Americans’ endorsement of Black stereotypes became less negative, and their endorsement of White American stereotypes became less positive (Hopkins & Washington, 2020). We acknowledge that causality cannot be established, however, speculate the crowd emotion amplification effect may be considered a form of implicit bias that is likely subject to sociocultural influences.

However, these explanations do not account for the observed interaction between race and trial size (1 face vs 12 faces). That is, why we observed a crowd emotion amplification effect for White faces, but an individual emotion amplification effect for Black faces. Much of this effect may be due to the rise in “White anger”, which is often expressed in real and online group settings by White people who feel xenophobic, disadvantaged, mistrustful, and fearful (e.g., Ganesh, 2020; Ott & Dickinson, 2020; Rudolph, 2021). A growing awareness of many Whites as angry may have led to an amplification effect that was greater for White crowds than Black crowds. More basic cognitive processes may also be at play. For instance, research suggests that race is perceived at an earlier stage of processing than emotional expression (Kubota & Ito, 2007), and that outgroup faces are processed in terms of race-relevant features at the expense of later individuating information such as emotional expressions (Levin, 2000). Consequently, for Black crowd trials participants’ ability to individuate may be impaired by early race categorisation, leading to judgements of lower anger intensity. Whereas, for White crowd trials this process may have facilitated greater individuation, facilitating integration of more “extreme” anger expressions into judgements of crowd emotion.

Readers may also wonder whether participant race influenced the observed results. In Experiments 1 and 3, no significant relationships between participant ethnicity and the crowd emotion amplification effect were observed. In Experiment 2, we observed a size × drink × crowd race × ethnicity interaction. White participants showed an increased amplification effect for White crowds compared to single faces but did not differ in their ratings of Black crowds compared to single faces. Other participants showed an increased amplification effect for White single faces compared to White crowds, regardless of alcohol prime. Other participants also showed an increased amplification effect for Black individuals compared to crowds, but only when preceded by alcohol primes. This finding may indicate that the crowd emotion amplification effect is not universal, but rather, sensitive to social and contextual factors.

#### Alcohol cues

Contrary to our prediction, alcohol cues did not reliably influence emotion amplification. Perhaps the presentation of alcohol cues for 1800 ms was insufficient to elicit an association strong enough to impact anger overestimation. Rather than using stimuli of people actually drinking alcohol, our manipulation was more subtle as it relied on priming alcoholic drinks. Previous research found that presenting alcohol-related images and words for 17 ms and 300 ms was sufficient to increase aggression and aggressive cognition relative to neutral stimuli (Subra et al., 2010). However, studies investigating the effects of alcohol cues in increasing hostile interpretations and the expression of racial bias have typically used longer cue priming tasks (Bartholow & Heinz, 2006; Stepanova et al., 2012). To our knowledge, these studies are the first to investigate the impact of alcoholic-cues on emotion perception in crowds. Future research should examine if longer exposure to alcohol primes and showing crowd members drinking alcohol can increase the crowd emotion amplification effect.

#### Gender

Contrary to our prediction, gender did not significantly influence the crowd emotion amplification effect. Participants overestimated anger in men more so than women, a finding that is consistent with the literature showing than men are typically perceived as angrier than women. However, the magnitude of the gender effect was not different for trials featuring individuals versus crowds. One possible explanation is that overestimation of men’s anger is equally applicable to individuals or crowds. Existing research challenges this explanation, although produces mixed findings. Research finds that for male anger bias in male faces was stronger for single trials than for crowd trials (Mihalache et al., 2021). However, research also finds that judgements of threat increased as the ratio of men to women in a group increased (Alt et al., 2019). Based on this latter evidence, anger bias in male faces should be stronger for crowd trials than for single trials. Another possibility is that additional features of the crowd setting may moderate the effect of gender on the emotion amplification effect. For example, presenting participants with crowds of men and women in traditionally masculine settings (e.g., military, sports teams, criminal gangs, construction workers) or feminine settings (e.g., nursing, day care centres) may affect crowd emotion amplification effects. Such work would be of theoretical and practical interest as it could identify situations that signal exaggerated threat from crowds of men.

### Limitations and future directions

The present research was limited in that the facial stimuli used for the crowd arrays comprised only a single identity. While the use of homogeneous face sets is a common methodological approach to control for differences between faces (e.g., Haberman & Whitney, 2007, 2009; Leib et al., 2012, 2014), it is not ecologically valid. Although our experiments were lacking in mundane realism (i.e., superficial similarity with the real-world), we suggest that they were high in psychological realism (i.e., eliciting the psychological processes thought to underlie the crowd emotional amplification effect). Nonetheless, it is plausible that crowds of single identities may be processed differently than crowds of multiple identities. A recent study found no difference in anger bias for crowds comprising single or multiple identities (Mihalache et al., 2021). However, set sizes contained a maximum of only four faces. It is possible that face homogeneity might impact crowd bias in larger sets such as those in the current studies.

Consistent with this idea, other evidence found ensemble coding accuracy was reduced when opportunities to encode individual identities were restricted (e.g., reduced time, increased set size; Neumann et al., 2018). Because opportunities to encode individual identities would be further restricted in crowds of multiple identities, this evidence would suggest that we would observe less accurate ensemble coding and thus greater anger amplification when using such stimuli. Thus, our sets of homogenous faces might have underestimated the true size of the crowd emotion amplification effect. Similarly, when viewing the crowd, participants may have initially gravitated towards the most extreme faces and then stopped processing them once participants realized they were identical. Experiments that combine artificial and real-world heterogeneous faces may provide insight into the boundary conditions and ecological validity of the present paradigm. For example, studies using eye tracking with mixed gender crowds may identify the extent to which men and women’s faces differentially contribute to anger overestimation.

Although replication suggests that the effects of our experimental manipulations are reliable, the effect sizes were small. We note that the largest effect in the model in terms of variance explained was the participant random effect; the effects of the experimental manipulations were much smaller. The crowd emotion amplification effect is thought to be mediated through visual attentional engagement, which results in a judgement of perceived anger via ensemble coding (Goldenberg et al., 2021). Although one would expect small effects of the manipulations on attentional processes, future research could benefit from stronger manipulations. Similarly, the relatively large proportion of variance accounted for by including participant as a random factor suggests that there are many unknown individual differences to be examined. We also note here the limitations of the samples used. On the one hand, we acknowledge the variability in sample characteristics between studies. That is, participants in Studies 1 and 2 were predominantly White and American, whereas participants in Study 3 were predominantly Asian and Australian. However, we also note that all samples were predominantly young, and residents in WEIRD countries, which may place additional constraints on the generalisability of the observed effects.

Another promising area of future research would be to manipulate the nature of the crowd environment. For instance, does the extent of anger amplification differ when crowds are portrayed as protestors versus concert goers? Answering questions like these could contribute to practical applications for personnel involved in crowd settings.

### Applied implications

This project has implications for crowd management and policing strategies. For example, recent social and political unrest has seen growing criticism of violent law enforcement responses to widespread protests in many parts of the world. When crowd control professionals (e.g., police, concert security) consider crowds with angry facial expressions as threatening, perceivers may be motivated to approach and confront the source of this threat (de Valk et al., 2015; Wilkowski & Meier, 2010). Alternatively, a crowd perceived as threating may elicit flight behaviour. Either outcome may result in injury to the crowd or crowd control personnel. As individuals tend to overestimate crowd anger, they may also be likely to respond disproportionately. Such actions are likely to escalate crowd hostility and aggression (Adang & Cuvelier, 2001).

Consequently, we recognize the need to design interventions that can minimize biases in attention and improve emotion perception in crowds. Doing so may improve safety for crowds and those who interact with them. Bias training could incorporate error management theory to explain to clients why people make this biased judgement. Crowds are not necessarily more angry than individuals or more threatening, but awareness of this bias could facilitate peaceful relations between crowds and the people who interact with them. By reducing the degree of perceived threat from crowds (as well as Black individuals and males), people may be better able to make decisions free of fear.

Reducing perceived threat may help reduce violence in the dynamic police-crowd interactions. A survey of 352 Italian police offers found that they endorsed using harsh punishments on crowds and failed to acknowledge any role for themselves in anti-social crowd behaviour (Prati & Pietrantoni, 2009). Similarly, bouncers can escalate violence through harassment and physical provocation (Roberts, 2009; Wells et al., 1998). These and other findings suggest there is a need for interventions to optimize responses to situations involving crowds or requiring crowd management. Our findings may be useful for interventions designed for this broad group of crowd professionals (e.g., police, bouncers, concert security, sporting event security). In sum, interventions would entail two processes; increasing awareness about the crowd emotion amplification effect, and education around gender, race, and alcohol.

## Conclusions

By independently replicating the crowd emotion amplification effect the current project provides additional evidence for the veracity of this phenomenon. We further identified that race (White versus Black) moderates the effect. Thus, the present research improves our understanding of how people use race to rapidly determine the emotional tone of crowds.

## Data Availability

The preregistration, data and R code are available at https://osf.io/2yr68/. Interested readers can view the full results, including non-significant results.

## Notes

1. Due to a technical error, we were unable to make exclusions based on reaction times as was stated in our preregistration. The reaction times recorded were highly inaccurate for some participants (e.g., negative or impossibly large values).

2. We made a minor coding error for the masks. Instead of presenting the mask for 300 ms on crowd trials, a blank screen was presented for 100 ms followed by a 200 ms mask for each face.

3. We also examined whether the effect of alcohol cues was moderated by self-reported drinking behaviour. This interaction was not significant in Experiment 1, *b* = −0.13, *SE* = .07, *t*(22338.00) = −1.94, *p* = .053; Experiment 2, *b* = −0.001, *SE* = .01, *t*(16985.49) = −0.05, *p* = .96; or Experiment 3, *b* = −.01, *SE* = .03, *t*(21389.01) = −0.48, *p* = .63.

4. We also examined whether the average anger intensity of each trial would interact with the experimental manipulation. In all experiments, average intensity was a strong predictor of the amplification effect. [Experiment 1: *b* = −0.27, *SE* = .04, *t*(22344.63) = −6.90, *p* < .0001; Experiment 2: *b* = −0.73, *SE* = .15, *t*(16909.71) = −4.92, *p* < .0001; Experiment 3: *b* = −0.68, *se* = .02, *t*(16980.71) = −38.17, *p* < .0001]. In Experiment 2, there was an additional mean intensity × race interaction, *b* = 0.07, *SE* = .03, *t*(16978.76) = 3.86, *p* = .0001. Post hocs controlling for size, gender, and drink showed that this effect of mean intensity on the amplification effect was significant for both black, = −0.68, *SE* = .01, *t*(8458.25) = −54.68, *p* < .0001, and white trials, *b* = −0.61, *SE* = .01, *t*(8420.84) = −48.29, *p* < .00000001, but was somewhat larger for Black trials. Key results remained significant (i.e., the main effects in Experiment 1 and interactions in Experiments 2 and 3). However, there were no interactions of average intensity with any of the remaining predictors.
